# Prevalence, trend and determinants of adolescent childbearing in Burundi: a multilevel analysis of the 1987 to 2016–17 Burundi Demographic and Health Surveys data

**DOI:** 10.1186/s12884-022-05009-y

**Published:** 2022-09-01

**Authors:** Jean Claude Nibaruta, Bella Kamana, Mohamed Chahboune, Milouda Chebabe, Saad Elmadani, Jack E. Turman, Morad Guennouni, Hakima Amor, Abdellatif Baali, Noureddine Elkhoudri

**Affiliations:** 1 Hassan First University of Settat, Higher Institute of Health Sciences, Laboratory of Health Sciences and Technologies, Settat, Morocco; 2 Hassan II University, Ibn Rochd University Hospital of Casablanca, Haematology laboratory, Casablanca, Morocco; 3 Indiana University, Richard M. Fairbanks School of Public Health, Departments of Social and Behavioral Sciences, Indianapolis, IN USA; 4 Cadi Ayyad University of Marrakech, Semlalia Faculty of Science, Departments of Biology, Marrakech, Morocco

**Keywords:** Adolescent, Childbearing, Determinants, Multilevel analysis, Burundi

## Abstract

**Background:**

Very little is known about factors influencing adolescent childbearing despite an upward trend in adolescent childbearing prevalence in Burundi, and its perceived implications on the rapid population growth and ill-health of young mothers and their babies. To adress this gap, this study aimed to examine the prevalence, trends and determinants of adolescent childbearing in Burundi.

**Methods:**

Secondary analyses of the 1987, 2010 and 2016–17 Burundi Demographic and Health Surveys **(**BDHS) data were conducted using STATA. Weighted samples of 731 (1987 BDHS), 2359 (2010 BDHS) and 3859 (2016-17BDHS) adolescent girls aged 15–19 years old were used for descriptive and trend analyses. Both bivariable and multivariable two-level logistic regression analyses were performed to identify the main factors associated with adolescent childbearing using only the 2016–17 BDHS data.

**Results:**

The prevalence of adolescent childbearing increased from 5.9% in 1987 to 8.3% in 2016/17. Factors such as adolescent girls aged 18–19 years old (aOR =5.85, 95% CI: 3.54–9.65, *p* <  0.001), adolescent illiteracy (aOR = 4.18, 95% CI: 1.88–9.30, *p* <  0.001), living in poor communities (aOR = 2.19, 95% CI: 1.03–4.64, *p* = 0.042), early marriage (aOR = 9.28, 95% CI: 3.11–27.65, *p* <  0.001), lack of knowledge of any contraceptive methods (aOR = 5.33, 95% CI: 1.48–19.16, *p* = 0.010), and non-use of modern contraceptive methods (aOR = 24.48, 95% CI: 9.80–61.14), *p* <  0.001) were associated with higher odds of adolescent childbearing. While factors such as living in the richest household index (aOR = 0.52, 95% IC: 0.45–0.87, *p* = 0.00), living in West region (aOR = 0.26, 95%CI: 0.08–0.86, *p* = 0.027) or in South region (aOR = 0.31, 95% CI: 0.10–0.96, *p* = 0.041) were associated with lower odds of adolescent childbearing.

**Conclusion:**

Our study found an upward trend in adolescent childbearing prevalence and there were significant variations in the odds of adolescent childbearing by some individual and community-level factors. School-and community-based intervention programs aimed at promoting girls’ education, improving socioeconomic status, knowledge and utilization of contraceptives and prevention of early marriage among adolescent girls is crucial to reduce adolescent childbearing in Burundi.

## Introduction

The World Health Organization (WHO) and United Nations entities define an adolescent as an individual aged 10–19 years [1, 2]. Adolescent childbearing is a major global public health issue because of its many adverse health and socio-economic consequences for both young mothers and their babies, particularly in Sub-Saharan Africa (SSA) [3, 4]. While adolescent childbearing declined significantly overall since 2004 [5], significant disparities persist between and within countries and among population groups, particularly in SSA [3, 6–8]. In 2015–2020, SSA had the highest levels of adolescent childbearing, followed by Asia and Latin America and the Caribbean [6]. Almost one-fifth (18.8%) of adolescent girls got pregnant in Africa, and a higher prevalence (21.5%) was observed in the East African sub-region where Burundi is located [3]. Several studies state that adolescent childbearing is associated with higher maternal mortality and morbidity and adverse child outcomes including a higher prevalence of low birth weight and higher perinatal and neonatal mortality as compared to older women [3, 4, 9]. Adolescent early initiation into childbearing lengthens the reproductive period and subsequently increases a woman’s lifetime fertility rate, contributing to rapid population growth [10–12].

The Burundian population is characterized by its extreme youth, with 65% under the age of 25 and almost a quarter of this growing population (23%) are adolescents [13]. In Burundi, adolescent childbearing remains an important issue because of its perceived implications on the rapid population growth and ill-health of adolescent mothers and their babies [11]. According to the report of the latest Burundi Demographic and Health Survey (BDHS) [14], 8% of women aged 15–19 begun childbearing, including 6% who had at least one live birth and 2% who were pregnant with their first child. Despite a good progress in reducing maternal mortality ratio [14], a large number of adolescent girls are still dying from pregnancy and childbirth related complications. The maternal mortality rate among Burundian adolescent girls is estimated at 150 maternal deaths per 1000 women aged 15–19 years [14]. Maternal disorders are the fourth highest cause of death among teenage mothers in Burundi [13]. Early marriage and adolescent pregnancy could lead to or aggravate anemia in mothers and result in low iron stores in the offspring [15], or in prematurity or low birth weight babies [16]. Approximately 36% of Burundian adolescent girls are anemic and 0.4% have obstetric fistula [14]. On the other hand, the infant mortality rate among adolescent girls in Burundi is estimated at 59 deaths per 1000 live births, of which 30% are neonatal and 29% post-neonatal [14]. In addition, the prevalence of low birth weight is higher among adolescent mothers (7.2%) than among women aged 20–34 years (4.7%) [14].

Several studies were conducted to examine the factors influencing adolescent pregnancy and motherhood in various settings. The results of these studies showed that early marriage or sexual intercourse [4, 7, 9], illiteracy or low level of education and poverty [3, 7, 9, 10] or living in poor neighborhoods [17, 18], age of the adolescent [4, 10, 19], marital status [3, 4, 10], rural residence and geographic regions [3, 4, 10, 20] are important factors influencing adolescent childbearing. Despite an upward trend in adolescent childbearing prevalence and its perceived implications on the rapid population growth and poor health of young mothers and their babies, very little is known about factors influencing adolescent childbearing in Burundi [21–23]. Only two BDHS reports [14, 24] containing information on factors influencing adolescent childbearing are available in Burundi. The results of these two surveys are limited to a few determinants of adolescent childbearing and are fully descriptive, and therefore do not make it possible to know the net effect of each of the factors influencing adolescent childbearing in the Burundian settings. To adress this gap, we aim to examine the prevalence, trend and determinants of adolescent childbearing using the 1987 to 2016–17 BDHS data.

## Data and methods

### Data sources and population

This study used adolescent women (aged 15–19) data extracted from the three BDHS conducted in 1987 [25], 2010 [24] and 2016–2017 [14] for descriptive statistics and the trend of adolescent childbearing assessment. For the second objective of identifying factors associated with adolescent childbearing, only adolescent women data from the most recent BDHS [14] were used. The BDHS are nationally representative surveys with samples based on a two-stage stratified sampling procedure: Enumeration areas or clusters in the first stage and households in the second stage. In sampled households, all women aged between 15 and 49 years who consent to participate in the survey are interviewed. Then 731, 2359, and 3859 adolescent women aged 15–19 years were successfully interviewed during the 1987, 2010 and 2016–17 BDHS surveys respectively. Thus, the current study used three weighted samples of 731, 2359, and 3859 adolescent women aged 15–19 years. A detailed description of the sampling procedure for each of these three surveys is presented in the final report for each survey [14, 24, 25].

### Variables of the study

#### Outcome variable

The outcome variable of interest in this study is adolescent childbearing, which refers to the sum of the percentage of adolescents aged 15–19 who are already mothers (have had at least a live birth) and the percentage of adolescents who are pregnant with their first child at the time of the interview [4, 26]. Thus, any adolescent who was already a mother or pregnant with her first child was coded one (1) and zero (0) in the opposite case.

#### Independent variables

Based on a prior literature review, our independent variables were classified into individual-level factors and community-level factors. The individual-level factors include: adolescent’s age, education, household wealth index, working status, religion, access to mass media, age at first marriage, knowledge of any contraceptive methods, and modern contraceptive use. Community-level factors include: place of residence, health regions, community-level education, and community-level poverty. It should be noted that of the four community-level variables, two variables (community-level education, and community-level poverty) were created by aggregating individual-level factors (adolescent’s education, and household wealth index) since these two variables are not directly found from the 2016–17 BDHS dataset.

#### Operational definitions

##### Access to mass media

Created by combining the following three variables: frequencies of listening to radio, watching TV, and reading newspapers and coded as “yes” if the adolescent was exposed to at least one of the three media and “no” in the opposite case.

##### Health regions

This variable had eighteen categories corresponding to the eighteen current provinces of Burundi. To reduce its excessive number of categories, it was recoded into five regions such as North Region, Central-East Region, West Region, South Region and Bujumbura Mairie [11].

##### Community-level education

Aggregate values measured by the proportion of adolescents with a minimum of primary level education derived from data on an adolescent’s education. Then, it was categorized using national median value to values: low (communities with *<* 50% of adolescents have at least primary education) and high (communities with ≥50% of adolescents have at least primary education) community-level of adolescent education.

##### Community-level poverty

Aggregate values measured by the proportion of adolescents living in households classified as poorest/poorer derived from data on household wealth index. Then, it was categorized using national median value to values: low (communities with *<* 50% of adolescents living in poorest/poorer households) and high (communities with ≥50% of adolescents living in poorest/poorer households) community-level of adolescent poverty.

### Data management and statistical analysis

After data were extracted, recoded and reorganized, the statistical analysis was performed using STATA statistical software version 14.2. During all statistical analyses, the weighted samples were used to adjust for non-proportional sample selection and for non responses to ensure that our results were nationally representative. Frequency and percentage were used to describe the sociodemographic characteristics as well as the sexual and reproductive health history of the sample across the three surveys. The trend analysis of adolescent childbearing was evaluated using the Extended Mantel-Haenszel chi square test for linear trend using the OpenEpi (Version 3.01)- Dose Response program [4, 27]. A *p*-value ≤0.05 was used to declare the existence of a significant trend.

During the BDHS data collection, two-stage stratified cluster sampling procedures were used and therefore the data were hierarchical. To obtain correct estimates in inferential analyses, advanced statistical models such as multilevel modeling that considers independent variables measured at individual- and community-levels should be used to account for the clustering effect/dependency [28–31]. Thus, bivariable and multivariable multilevel logistic regression analyses were conducted to identify factors associated with adolescent childbearing by using only the most recent BDHS [14]. We first performed the bivariable multilevel logistic regression analysis to examine associations between adolescent childbearing and the selected individual and community-level variables. Then variables with a *p*-value ≤0.2 in the bivariate analysis were included in the multivariable multilevel logistic regression analysis to assess the net effects of each independent variable on adolescent childbearing after adjusting for potential confounders. The fixed effects were reported in terms of adjusted odds ratios (aOR) with 95% confidence intervals (CI) and *p*-values. Variables with *p*-value < 0.05 were declared to be significantly associated with adolescent childbearing in the multivariate analysis.

Before performing these multilevel logistic regression analyses, an empty model was conducted to calculate the extent of variability in adolescent childbearing between clusters (between communities). The existence of this variability was assessed using the Intra-Class correlation Coefficient (ICC) and the Median Odds Ratio (MOR) [29–32]. The ICC represents the proportion of the between-cluster variation in the total variation (the between- plus the within-Cluster variation) of the chances of adolescent childbearing [28, 29]. It can be computed with the following formula:

$${\displaystyle \begin{array}{c}\mathrm{ICC}={\sigma}^2/\left({\sigma}^2+{\pi}^2/3\right)\\ {}.=\frac{\sigma^2}{\sigma^2+3.29},\mathrm{where}\ {\sigma}^2\mathrm{represents}\ \mathrm{the}\ \mathrm{cluster}\ \mathrm{variance}\end{array}}$$

The MOR is the Median values of the Odds Ratio of the cluster at high risk and cluster at lower risk of adolescent childbearing when randomly picking two adolescent women from two different clusters [29, 30] . It can be computed with the following formula:

$$\mathrm{MOR}=\exp\ \left[\sqrt{\Big(2\times {\sigma}^2}\right)\times 0.6745\Big]$$

$$\mathrm{MOR}\cong \exp\ \left(0.95\times \sqrt{\sigma^2}\right)$$

The deviance (or-2Log likelihood), Akaike Information Criteria (AIC) and Bayesian Information Criterion (BIC) were used to compare the fit to the data of the null model and that of the full model where we favored model with smaller values of these indices [4, 30, 33].

## Results

### Sociodemographic characteristics of samples

The sociodemographic characteristics of the adolescents included in the three surveys are summarized in Table 1. The analysis of adolescents’ age showed that the majority of them (53.4, 61.1 and 64.5% in the 1987, 2010 and 2016–17 BDHS respectively) were between 15 and 17 years old. Similarly, most of participants resided in rural areas: 95.7% (1987 BDHS), 88.4% (2010 BDHS) and 85.8% (2016–17 BDHS). A large proportion of adolescents (75.8 and 76.5% in the 2010 and 2016–17 BDHS respectively) lived in three health regions (North, Central-East and South). Similarly, most adolescent girls were still single: 93.2% (1987 BDHS), 90.2% (2010 BDHS) and 93.3% (2016–17 BDHS). The proportion of illiterate adolescents decreased from 73.3% (1987 BDHS) to 7.3% (2016–17 BDHS). On the other hand, the percentages of adolescents who were currently working increased from 7.5% (1987 DHS) to 57.6% (2016-17DHS). More than half of adolescent girls (58.5 and 53.6% in the 2010 and 2016–17 BDHS surveys respectively) were from very poor/poor/middle-income households. Similarly, analysis of religious affiliation showed that most adolescents were Catholic: 61.1% (2010 BDHS) and 55.7% (2016–17 BDHS).

**Table 1:** Sociodemographic characteristics of adolescents in Burundi using the 1987, 2010 and 2016/17 BDHS

Variables / categories	BDHS year		
	1987 (***N*** = 731)	2010(***N*** = 2359)	2016–17(***N*** = 3859)
	n (%)	n (%)	n (%)
**Adolescent’s Age**			
15–17 years	390 (53.4)	1442 (61.1)	2489 (64.5)
18–19 years	341 (46.6)	917 (38.9)	1370 (35.5)
**Residence**			
Rural	700 (95.7)	2087 (88.4)	3310 (85.8)
Urban	31 (4.3)	273 (11.6)	548 (14.2)
**Health Regions**			
Bujumbura Mairie		193 (8.2)	315 (8.2)
North		657 (27.8)	1046 (27.1)
Central East		565 (23.9)	947 (24.5)
West		378 (16.0)	589 (15.3)
South		568 (24.1)	961 (24.9)
**Marital Status**			
Single	682 (93.2)	2128 (90.2)	3601 (93.3)
Married/living together	43 (5.9)	201 (8.5)	227 (5.9)
Divorced/separated/widowed	6 (0.8)	30 (1.3)	31 (0.8)
**Education**			
No education	536 (73.3)	500 (21.2)	281 (7.3)
Primary	186 (25.4)	1425 (60.4)	1836 (47.6)
Secondary and above	9 (1.2)	434 (18.4)	1742 (45.1)
**Currently working**			
No	677 (92.5)	1112 (47.1)	1634 (42.4)
Yes	54 (7.5)	1248 (52.9)	2224 (57.6)
**Wealth Quantile**			
Poorest		444 (18.8)	589 (15.3)
Poorer		469 (19.9)	711 (18.4)
Middle		468 (19.8)	769 (19.9)
Richer		453 (19.2)	840 (21.8)
Richest		525 (22.2)	950 (24.6)
**Religion**			
No religion		12 (0.5)	10 (0.3)
Catholic		1442 (61.1)	2147 (55.7)
Protestant		792 (33.5)	1417 (36.7)
Adventist		43 (1.8)	124 (3.2)
Muslim		50 (2.1)	105 (2.7)
Others^a^		22 (0.9)	56 (1.5)

Others

^a^Jehovah witness and other sects

### Sexual and reproductive health characteristics of the samples

The percentage of adolescents who had their first sexual intercourse at age ≤ 14 years increased from 0.7% (1987 BDHS) to 2.6% (2016–17 BDHS). Similarly, the percentage of adolescents who had their first birth at age ≤ 17 years increased from 1.7% (1987 BDHS) to 3.3% (2016–17 BDHS). In contrast, the proportion of adolescents who had their first marriage at age ≤ 17 decreased slightly from 4% (1987 BDHS) to 3.8% (2016–17 BDHS). Similarly, 40.1% (1987 BDHS) of adolescents had knowledge of any contraceptive methods compared to 89.9% (2016–17 BDHS). The percentage of adolescents who do not intend to use contraception increased from 17.8% (2010 BHDS) to 24.8% (2016–17 BDHS). On the other hand, there was a reduction in the proportion of adolescents with unmet need for contraception, which decreased from 3.2% (2010 BDHS) to 2.5% (2016–17 BDHS). Regarding fertility preference, 5.8% (2010 BDHS) of adolescents wanted to have another pregnancy compared to 96.5% in the 2016–17 BDHS (See Table 2).

**Table 2:** Sexual and reproductive health characteristics of adolescents in Burundi using the 1987, 2010 and 2016/17 BDHS data

Variables /categories	BDHS year		
	1987 (***N*** = 731)	2010(***N*** = 2359)	2016–17(***N*** = 3859)
	n (%)	n (%)	n (%)
**Age at First Sex**			
≤ 14 years	5 (0.7)	83 (3.5)	101 (2.6)
15–17 years	32 (4.4)	217 (9.2)	326 (8.5)
18–19 years	24 (3.3)	74 (3.2)	139 (3.6)
Not had sex/inconsistent/missing	670 (91.6)	1985 (84.1)	3292 (85.3)
**Age at First Marriage**			
≤ 17 years	29 (4.0)	159 (6.7)	148 (3.8)
18–19 years	20 (2.8)	72 (3.1)	109 (2.8)
Never in union	682 (93.2)	2128 (90.2)	3601 (93.3)
**Age at First Birth**			
≤ 17 years	13 (1.7)	84 (3.6)	127 (3.3)
18–19 years	11 (1.5)	74 (3.1)	107 (2.8)
Still no birth	708 (96.8)	2201 (93.3)	3624 (93.9)
**Knowledge of Any Contraceptive Methods**			
Has knowledge	293 (40.1)	2166 (91.8)	3468 (89.9)
No knowledge	438 (59,9)	193 (8.2)	390 (10.1)
**Contraceptive Use and Intention**			
Using modern method		30 (1.3)	98 (2.5)
Using traditional method		4 (0.2)	18 (0.5)
Non-user - intends to use later		1904 (80.7)	2785 (72.2)
Does not intend to use		421 (17.8)	958 (24.8)
**Unmet Need for Contraception**			
Never had sex		1688 (71.5)	2,878 (74.6)
Unmet need for spacing/limiting		75 (3.2)	95 (2.5)
Using for spacing/liming		34 (1.5)	116 (3.0)
No unmet need		154 (6.5)	161 (4.2)
Not married and no sex in last 30 days		104 (4.4)	195 (5.1)
Infecund/menopausal		304 (12.9)	413 (10.7)
**Fertility Preference**			
Have another pregnancy	42 (5.8)	2254 (95.5)	3724 (96.5)
Undecided	0 (0,0)	49 (2.1)	33 (0.8)
No more	0 (0.1)	31 (1.3)	77 (2.0)
Declared infecund/Sterilized	–	24 (1.0)	25 (0.7)
Missing	688 (94 .1)	2 (0.1)	–

### Prevalence and trends of adolescent childbearing

**Fig. 1:**
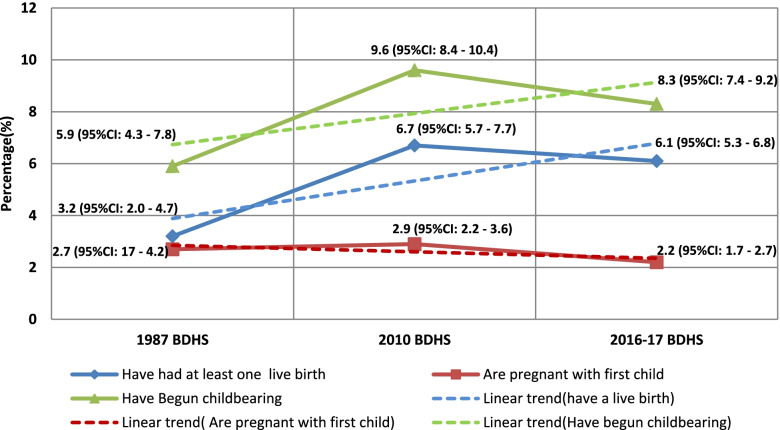
Prevalence and trends of adolescent childbearing in Burundi using the 1987, 2010 and 2016–17 BDHS Data

The prevalence and trends of adolescent childbearing were examined in its two components: prevalence and trend of adolescents who have had at least one live birth and prevalence and trend of those who were pregnant with their first child at the time of the survey (see Fig. 1). Thus, the prevalence of adolescent childbearing increased from 5.9% (95% CI: 4.3–7.8) in 1987 to 9.6% (95% CI: 8.4–10.4) in 2010, and then decreased from 9.6 to 8.3% (95% CI: 7.4–9.2) in 2016/17. The trend analysis shows that there was an increase of 2.4% from 1987 to 2016/17 although this increase was not statistically significant (*P*-value = 0.0503). Indeed, the prevalence of adolescents who have had at least one live birth increased from 3.2% (95% CI: 2.0–4.7) in 1987 to 6.7% (95% CI: 5.7–7.7) in 2010, and then decreased from 6.7 to 6.1% (95% CI: 5.3–6.8) in 2016/17. The trend analysis shows that there was an increase of 2.9% from 1987 to 2016/17 and this increase was statistically significant (*P*-value = 0.0036). On the other hand, the prevalence of adolescents who were pregnant with their first child increased from 2.7% (95% CI: 1.7–4.2) in 1987 to 2.9% (95% CI: 2.2–3.6) in 2010, and then decreased from 2.9 to 2.2% (95%CI: 1.7–2.7) in 2016/17. The trend analysis shows that there was a decrease of 0.5% from 1987 to 2016/17 but this decrease was not statistically significant (*P*-value = 0.3593).

### Determinants of adolescents childbearing

Bivariable and multivariable multilevel logistic regression analyses were conducted to identify individual and community-level factors associated with adolescent childbearing by using only the most recent (2016–17) BDHS data. First, an empty model was performed to calculate the extent of variability in adolescent childbearing between clusters by using the ICC and the MOR indicators. The deviance, AIC, and BIC were also used to select the model that best fit the data. The results of bivariable and multivariable analyses, random effect model and model fitness are summarized in Table 3.

**Table 3:** Results of bivariable and multivariable multilevel logistic regression analyses of factors associated with adolescent childbearing in Burundi

Variables/categories	2016–17 BDHS (weighted sample, ***N*** = 3859)			
	Bivariable analysis		Multivariable analysis	
	uOR (95%CI)	***P*** value	aOR (95% CI)	***P*** value
***Individual-level variables***				
**Adolescent’s age**				
15–17 years (RC)	1.00		1.00	
18–19 years	13.87 (9.93–19.38)	< 0.001	5.85 (3.54–9.65)	**< 0.001**
**Education**				
Secondary /High (RC)	1.00		1.00	
Primary	2.71 (2.02–3.64)	< 0.001	2.58 (1.54–4.25)	**< 0.001**
No education	5.79 (3.80–8.80)	< 0.001	4.18 (1.88–9.30)	**< 0.001**
**Currently Working**				
No (RC)	1.00		1.00	
Yes	3.17 (2.34–4.29)	< 0.001	1.43 (0.86–2.39)	0.170
**Household Wealth Index**				
Poorest (RC)	1.00		1.00	
Poorer	0.56 (0.38–0.84)	0.005	1.18 (0.56–2.46)	0.660
Middle	0.50 (0.34–0.75)	0.001	0.75 (0.34–1.12)	0.467
Richer	0.56 (0.38–0.83)	0.004	0.61 (0.55–1.07)	0.052
Richest	0.46 (0.31–0.70)	< 0.001	0.52 (0.45–0.87)	**0.007**
**Religion**				
No religion (RC)	1.00		1.00	
Catholic	0.21 (0.04–1.05)	0.057	2.78 (0.02–495.32)	0.698
Protestant	0.28 (0.05–1.41)	0.122	2.98 (0.02–531.20)	0.679
Adventist	0.60 (0.11–3.30)	0.559	3.25 (0.02–653.55)	0.663
Muslim	0.40 (0.07–2.27)	0.303	10.55 (0.06–2016.49)	0.379
Others^**a**^	0.44 (0.07–2.73)	0.381	4.22 (0.02–960.45)	0.603
**Access to Mass Media**				
No Access (RC)	1.00		1.00	
Has Access	0.65 (0.51–0.84)	0.001	0.67 (0.43–1.06)	0.090
**Age at First Marriage**				
18–19 years (RC)	1.00		1.00	
≤ 17 years	9.66 (3.57–26.11)	< 0.001	9.28 (3.11–27.65)	**< 0.001**
Never in union (NA)	0.00 (0.00–0.01)	< 0.001	0.01 (0.00–0.02)	**< 0.001**
**Knowledge of Any Contraceptive Methods**	1.00			
Has Knowledge (RC)	1.00		1.00	
No Knowledge	9.86 (3.82–25.44)	< 0.001	5.33 (1.48–19.16)	**0.010**
**Modern Contraceptive Use**				
Yes (RC)	1.00		1.00	
No	35.17 (21.03–58.79)	< 0.001	24.48 (9.80–61.14)	**< 0.001**
***Community-Level Variables***				
**Place of Residence**				
Rural (RC)	1.00			
Urban	1.17 (0.77–1.78)	0.456		
**Health Regions**				
Bujumbura Mairie	1.00		1.00	
North	0.96 (0.54–1.70)	0.887	0.46 (0.14–1.44)	0.182
Central East	0.56 (0.31–1.02)	0.058	0.34 (0.11–1.05)	0.062
West	0.83 (0.44–1.54)	0.546	0.26 (0.08–0.86)	**0.027**
South	0.44 (0.24–0.81)	0.008	0.31 (0.10–0.96)	**0.041**
**Community-level Education**				
Low (RC)	1.00			
High	0.88 (0.60–1.27)	0.491		
**Community-level Poverty**				
Low (RC)	1.00		1.00	
High	1.31 (0.97–1.78)	0.083	2.19 (1.03–4.64)	**0.042**
**Random effect**	Empty model		Full model	
Community variance (SE)	0.83 (0.20)		0.62 (0.14)	
ICC (%)	20.2		15.8	
MOR	2.37		2.11	
**Model fitness**				
Log likelihood	− 1079.61		− 452.85	
Deviance	2159.22		905.70	
AIC	2163.22		955.71	
BIC	2175.74		1112.16	
**Clusters**	553		553	

Note: Others

^a^Jehovah witness and other sects

*AIC* Akaike’s Information Criterion, *BIC* Bayesian Information Criterion, *ICC* Intra-Cluster Correlation, *MOR* Median Odds Ratio, *SE* Standard Error, *uOR* Unadjusted Odds Ratio, *aOR* Adjusted Odds Ratio, *95% CI* 95% Confidence Interval

According to the findings in Table 3, the ICC of the empty model was estimated to 20.2, which indicated that about 20.2% of the variations in adolescent childbearing were attributable to community differences. Similarly, the MOR of the empty model was estimated to 2.37, which means that if we randomly selected two adolescent girls from two different communities, the one from a higher risk community had 2.37 times higher odds of childbearing than the one from a lower risk community. The model fitness findings revealed the best-fitted model was the full model (model with individual and community-level factors) since it had significantly (*p* <  0.001) lower values of deviance (905.70), AIC (955.71), and BIC (1112.16) compared to those of the empty model. In the bivariable analysis, factors like adolescent’s age, education, working status, household wealth index, religion, access to mass media, age at first marriage, knowledge of any contraceptive methods, modern contraceptive use, health regions and community-level poverty met the minimum criteria (*p ≤* 0.2) to be included in the multivariable analysis.

In the multivariable analysis, only factors such as adolescent’s age, adolescent’s education, household wealth index, age at first marriage, knowledge of any contraceptive methods, modern contraceptive use, health regions, and community-level poverty remained significantly associated with adolescent childbearing. Indeed, adolescents aged 18–19 years had about 6 times higher odds (aOR =5.85, 95% CI: 3.54–9.65, *p* <  0.001) of childbearing than those aged 15–17 years. The odds of childbearing among adolescents who had no education was about 4 times higher (aOR = 4.18, 95% CI: 1.88–9.30, *p* <  0.001), and those who had only a primary education was about 2 times higher (aOR = 2.58, 95% CI: 1.54–4.25, *p* <  0.001) than adolescents who had a secondary or high education. The adolescents in the richest household quintile had 48% lower odds (aOR = 0.52, 95% IC: 0.45–0.87, *p* = 0.007) of childbearing compared to those in the poorest household quintile.

Similarly, the odds of childbearing among adolescents who got married at ≤17 years old was about 9 times higher (aOR = 9.28, 95% CI: 3.11–27.65, *p* <  0.001) than those who got married at the age between 18 and 19. Moreover, the adolescents who didn’t have knowledge of any contraceptive methods had about 5 times higher odds (aOR = 5.33, 95% CI: 1.48–19.16, *p* = 0.010) of childbearing than those who had knowledge of any contraceptive methods. Similarly, the odds of childbearing among adolescents who were not using modern contraceptive methods was about 24 times higher (aOR = 24.48, 95% CI: 9.80–61.14), *p* <  0.001) than those who were using modern contraceptive methods. Also, the odds of childbearing among adolescents living in West, and those in South were about 74% (aOR = 0.26, 95%CI: 0.08–0.86), *p* = 0.027) and 69% (aOR = 0.31, 95% CI: 0.10–0.96, *p* = 0.041) times lower respectively than those living in Bujumbura Mairie. Finally, the odds of childbearing among adolescents living in high community-level poverty was about 2 times higher (aOR = 2.19, 95% CI: 1.03–4.64, *p* = 0.042) than those living in low community-level poverty**.**

## Discussion

This study aimed to analyze the prevalence, trend and determinants of adolescent childbearing in Burundi using data from the three DHS conducted in Burundi in 1987 [25], 2010 [24], and 2016–17 [14] respectively. Our findings showed that the prevalence of adolescent childbearing increased from 5.9% in 1987 to 8.3% in 2016/17. Indeed, analysis of the trend in adolescent childbearing over a 30-year period (1987 to 2017) shows that there was an increase in adolescent childbearing between 1987 and 2010, which would likely be the result of the various consequences of the 1993–2005 civil war. These consequences include sexual violence [34], the increase in the poverty rate [13, 35, 36] and the gradual deterioration of social norms that prohibited pregnancy outside of marriage, especially in urban areas [37]. Afterwards, there was a slight decrease in adolescent childbearing between 2010 and 2017, which would be attributable to the general increase in education in Burundi since 1987 but especially since 2010 after the implementation of the free Primary School Policy (FPSP) by the Burundian government in 2005 [38]. However, the effect of this general increase in school enrollment (at the individual and especially at the community level) would have been mitigated by the increase in the poverty rate among households especially after the 2015 post-election crisis [39] as some girls opt for early marriage to escape the poor household conditions in the parental home [35], while others move alone to the cities, especially in Bujumbura Mairie, in search of work and are often vulnerable to sexual exploitation which puts them at high risk of becoming pregnant [34], the gradual deterioration of social norms that severely prohibited pregnancy outside of marriage especially in urban areas [37], and finally the difficulties of access/low utilization of family planning services by adolescents girls in Burundi [23, 40, 41]. Although this upward trend in adolescent childbearing was not statistically significant, Burundi should make greater efforts to reverse this trend given the negative impact of adolescent childbearing in Burundi on the young mothers and their babies’ well-being [21, 34, 42] and on the current demographic pressure [11, 13]. Moreover, several studies showed that the high level of maternal and infant morbidity and mortality can be reduced by reducing the adolescent childbearing rates in developing countries [3, 43, 44]. In addition, Burundi should take as a good example most of its neighboring countries that are currently showing a downward trend in adolescent childbearing after having made enormous efforts [4, 7].

Our study identified some key determinants of adolescent childbearing in the Burundian settings. Indeed, our findings indicated that adolescents aged 18–19 years were more likely to start childbearing than those aged 15–17 years. This positive correlation between adolescent age and risk of childbearing could be explained by increased exposure to sexual intercourse and marriage as the age of adolescent increases [4, 10]. Our results are consistent with those of many previous studies [4, 7, 10] that showed that the odd of adolescent pregnancy increases with adolescent age.. However, it should be noted that the consequences of childbearing can be much more serious for 15–17 year old girls than for 18–19 year old girls, both in terms of their health (given their physical immaturity) and that of their babies, in terms of acceptance in the community given that the legal age of marriage in Burundi is 18, and in terms of an increase in their reproductive age which would contribute to a high fertility rate further exacerbating the demographic pressure in Burundi [11]. Therefore, intervention programs to reduce/prevent adolescent childbearing in Burundi should preferably target all age groups of adolescent girls.

Similarly, our results showed that adolescents who had no education were more likely to start childbearing than those who had a secondary or high education. Such an association could be explained by the fact that out-of-school adolescent girls do not have access to comprehensive sexuality education (CSE) [45] and skills necessary to negotiate sexuality and reproductive options [3]. The protective effect of education against adolescent childbearing has also been reported in several previous studies. Indeed, adolescents who had no education had about 2 times higher odds of childbearing compared to those who were in school [3]. Teenage girls who had no education had about 3 times higher odds of childbearing than those who had a secondary or high education [45] . Other similar results were reported in studies conducted in Malawi [10], and in five East African countries that do not include Burundi [7]. In Burundi, a significant increase in the school attendance rate, especially at the primary level, was observed following the implementation of the FPSP initiated by the Burundian government since 2005 [38]. However, there is still a gender gap in school attendance, especially at the secondary and higher levels [14, 38]. Moreover, CSE was certainly integrated into the education program in Burundi even in extracurricular school clubs [22]. However, this is not enough as the emphasis was placed on abstinence as the only accepted method for avoiding adolescent pregnancy [37, 38]. The information available on the benefits of using contraceptive methods would be also very limited to have a positive effect on girls’ possibilities to protect themselves [22]. Furthermore, many adolescent girls are eventually forced to drop out of school because of the very poor living conditions in the parental home [35, 36] and face an increased risk of pregnancy while trying to provide for their basic needs themselves [34, 35, 38]. Given the importance of education, particularly at the secondary and tertiary levels, in preventing teenage childbearing, policymakers should do everything possible to promote young girls education at all levels of the Burundian education system while significantly improving the household socio-economic conditions and the quality of the CSE provided.

Our findings also revealed that household poverty or living in poor communities is associated with higher odds of adolescent childbearing. In the Burundian context, this association could be explained by the fact that Burundian society was highly affected economically by the civil war of 1993–2005 [34, 37]. Consequently, 64.9% of Burundians live below the national poverty line of US$1.27 and 38.7% live in extreme poverty [35, 36]. Thus, some rural adolescents arrive alone in cities in search of work and are often vulnerable to sexual exploitation, which exposes them to a high risk of unwanted pregnancies [34, 38]. On the other hand, some adolescent girls, especially those from rural areas, are eventually forced to drop out of school, either because they have no money to buy sanitary pads during menstruation or because they are unable to learn much without some food before school or at lunchtime [38]. Some malicious men (shopkeepers, drivers, teachers, etc.) take advantage of this precariousness to offer them money in exchange for sex, which often results in unwanted pregnancies [13, 22].. Our results corroborate those of the study by Vikat et al. [17] and those of the study by Kearney and his colleague [18]. Although the relationship between poverty and adolescent childbearing may be a vicious cycle [3], our findings and available evidence [7, 9, 13] underscore the importance of improving the households’ socioeconomic status in general, but especially of disadvantaged communities, to reduce the prevalence of adolescent childbearing, thereby improving their sexual and reproductive health.

Unexpectedly, Bujumbura Mairie, which is generally considered less poor than other regions and where more youth have access to education [38], was found to be associated with a higher risk of adolescent pregnancy than other regions. This finding could be explained by two main reasons. The first is that in order to escape poor living conditions in parental households, some rural adolescents arrive alone in Bujumbura Mairie in search of work and are often vulnerable to sexual exploitation, which puts them at increased risk of becoming pregnant [34]. The second reason is that rural families are even more attached to social norms against out-of-wedlock pregnancies than urban families [34, 37]. Therefore, to escape the stigma of their families, some rural adolescents who experience an unwanted pregnancy prefer to move to Bujumbura Mairie as soon as possible before the family realizes that their daughter is pregnant.

This study also found that the adolescent early marriage is associated with a higher odd of childbearing. This link between early marriage and higher risk of adolescent childbearing could be justified by the fact that early marriage implies early sexual debut and therefore a major risk of early pregnancy and childbearing [7, 9, 46]. In addition, several previous studies [3, 4, 9, 46] reported similar results. In Burundi, early marriage is associated with not only young mothers’ and their babies’ poor health outcomes [14], but also with high fertility rate [11]. While the official age of marriage for girls in Burundi is 18, early marriage remains a common practice, especially in rural areas, as a way to escape poor living conditions in the parental home [35]. Therefore, the Burundian government should ensure the strict enforcement of any law aimed at combating early marriage while improving the socio-economic conditions of households. Indeed, apart from the findings of our study, several other researchers [3, 4, 46, 47] suggest that investing in the prevention of child marriage is important not only to reduce teenage pregnancies and related complications, but also to improve a country’s economic development.

Similarly, our findings showed that both the lack of knowledge of any contraceptive methods and the non-use of modern contraceptive methods were associated with higher odds of adolescent childbearing. The positive influence of good knowledge and use of family planning services in preventing or reducing the rate of unintended pregnancies among adolescent girls has been widely reported in the scientific literature [9, 10, 42, 46]. However, most Burundian adolescent girls do not use contraception, and some do not even plan to use it in the future [14]. Indeed, the prevalence of contraceptive use among adolescent girls remains very low (2.5%) and the percentage of adolescents girls who do not intend to use contraception increased from 17.8% in 2010 to 24.8% in 2016–17. Moreover, the percentage of adolescents who had knowledge of any contraceptive methods decreased from 91.8% in 2010 to 89.9% in 2016–17 [14, 24]. The results of this study as well as the available evidence [46, 47] highlight the importance of interventions such as CSE [42] at all levels of the Burundian education system and provision of contraceptive services [48] to adolescents and creating supportive environments such as knowledge and support from parents, teachers, church, mass media campaign, governance, and a peer education program [42, 46] to reduce the prevalence of adolescent childbearing in Burundi. The strength of our study is that it would be among the first to focus on trend analyses and community-level factors in the analysis of determinants of adolescent childbearing in Burundi. In addition, this study is the first to use an advanced logistic regression model (multilevel model) to investigate the determinants of adolescent childbearing in Burundi. However, our study also suffers from some limitations. The 1987 DHS database did not contain some of the variables of interest to our study. Therefore, we limited ourselves to the analysis of the available variables. Moreover, the results of this study may suffer from misreporting bias regarding the respondents current ages. Indeed, respondents’ ages may not always have been reported correctly, either intentionally by trying to report a higher age than the real age given the stigma surrounding adolescent pregnancy [21] and the legal consequences of early marriage, or by not knowing the real age given that Burundi has suffered from repeated outbreaks of mass violence and political crisis [34, 37] during which registration of birth dates in government records was often impossible [49]. In addition, our study looked only at current pregnancies or previous births of adolescents to assess the prevalence of adolescent childbearing and did not consider adolescent pregnancies that ended in miscarriage, abortion, or stillbirth. This consideration is very important in the interpretation of the results of this study by readers, as there may be an underestimation bias in the prevalence. Indeed, given the Burundian culture, which still considers pregnancy outside of marriage to be a disgrace to the family [21], many cases of induced and clandestine abortion are quite possible in Burundi, as was found in two recent studies conducted in two of Burundi’s neighboring countries, in Uganda [50] and in Ethiopia [51], which showed that nearly one in six adolescent pregnancies ends in an induced and clandestine abortion. Further studies that include adolescent pregnancies that ended in miscarriage, abortion, or stillbirth in prevalence estimate are needed to better understand the extent of the problem in Burundi.

## Conclusion

The prevalence of adolescent childbearing increased from 5.9% in 1987 to 8.3% in 2016/17 although this increase was not statistically significant. There were variations in the odds of adolescent childbearing by some individual and community-level factors. Factors such as late adolescent age, adolescent illiteracy, household poverty or high community-level poverty, early marriage, lack of knowledge of any contraceptive methods, non-use of modern contraceptive methods, and living in Bujumbura Mairie were associated with higher odds of adolescent childbearing. School- and community- based intervention programs aimed at promoting girls’ education and improving socioeconomic status, knowledge and utilization of contraceptives and prevention of early marriage among adolescent girls is crucial to reduce adolescent childbearing in Burundi.

## Data Availability

The data that support the findings of this study are available for download upon a formal application from the DHS Program web site https://dhsprogram.com/data/available-datasets.cfm, but restrictions apply to the availability of these data, which were used under license for the current study, and so are not publicly available. Data are however available from the authors upon reasonable request and with permission of the DHS Program.

## Abbreviations

AIC: Akaike Information Criterion
aOR: Adjusted Odds Ratio
BDHS: Burundi Demographic and Health Survey
BIC: Bayesian Information Criterion
CSE: Comprehensive sexuality Education
FPSP: Free Primary Schooling Policy
ICC: Intra-Class correlation Coefficient
MOR: Median Odds Ratio
SSA: Sub-Saharan Africa
WHO: World Health Organization

## References

[1] WHO (2018). Guidance on ethical considerations in planning and reviewing research studies on sexual and reproductive.pdf.

[2] Plummer ML, Baltag V, Strong K, Dick B, Ross DA, World Health Organization (2017). Global Accelerated Action for the Health of Adolescents (AA-HA!): guidance to support country implementation.

[3] Kassa GM, Arowojolu AO, Odukogbe AA, Yalew AW (2018). Prevalence and determinants of adolescent pregnancy in Africa: a systematic review and Meta-analysis. Reprod Health.

[4] Kassa GM, Arowojolu AO, Odukogbe A-TA, Yalew AW (2019). Trends and determinants of teenage childbearing in Ethiopia: evidence from the 2000 to 2016 demographic and health surveys. Ital J Pediatr.

[5] World Bank, International Monetary Fund. Global Monitoring Report 2015/2016: Development Goals in an Era of Demographic Change. Washington, DC: World Bank; 2016.

[6] United Nations (2019). World Fertility 2019 : early and later childbearing among adolescent women (ST/ESA/SER.A/446).

[7] Wado YD, Sully EA, Mumah JN. Pregnancy and early motherhood among adolescents in five East African countries: a multi-level analysis of risk and protective factors. BMC Pregnancy Childbirth. 2019;19:59. Available on: https://bmcpregnancychildbirth.biomedcentral.com/articles/10.1186/s12884-019-2204-z. 10.1186/s12884-019-2204-z PMC6366026 30727995

[8] WHO. Adolescent pregnancy. Available from: https://www.who.int/news-room/fact-sheets/detail/adolescent-pregnancy. Cited 2021 Jan 12

[9] World Health Organization. Regional Office for South-East Asia. Adolescent pregnancy situation in South-East Asia Region. Geneva: World Health Organization; 2015.

[10] Palamuleni ME (2017). Determinants of adolescent fertility in Malawi. Gend Behav..

[11] Nibaruta JC, Elkhoudri N, Chahboune M, Chebabe M, Elmadani S, Baali A, et al. Determinants of fertility differentials in Burundi: evidence from the 2016–17 Burundi demographic and health survey. PAMJ. 2021;38 Available from: https://www.panafrican-med-journal.com/content/article/38/316/full. Cited 2021 Apr 2. 10.11604/pamj.2021.38.316.27649 PMC8265263 34285739

[12] Islam MM (1999). Adolescent childbearing in Bangladesh. Asia Pacific Population Journal.

[13] Rasmussen B, Sheehan P, Sweeny K, Symons J, Maharaj N, Kumnick M, et al. Adolescent Investment Case in Burundi: Estimating the Impacts of Social Sector Investments for adolescents. Bujumbura: Burundi: UNICEF Burundi; 2019.

[14] Ministère à la Présidence chargé de la Bonne Gouvernance et du Plan (MPBGP), Ministère de la Santé Publique et de la Lutte Contre le Sida (MSPLS), Institut de Statistiques et d’Études Économiques du Burundi (ISTEEBU), ICF. Troisième Enquête Démographique et de Santé 2016–2017. Bujumbura, Burundi: ISTEEBU, MSPLS, and ICF.; 2017. 679. Available from: https://dhsprogram.com/publications/publication-FR335-DHS-Final-Reports.cfm

[15] Kalaivani K (2009). Prevalence & consequences of anaemia in pregnancy. Indian J Med Res Citeseer.

[16] Ahmad MO, Kalsoom U, Sughra U, Hadi U, Imran M (2011). Effect of maternal anaemia on birth weight. J Ayub Med Coll Abbottabad.

[17] Vikat A, Rimpelä A, Kosunen E, Rimpelä M (2002). Sociodemographic differences in the occurrence of teenage pregnancies in Finland in 1987–1998: a follow up study. J Epidemiol Community Health.

[18] Kearney MS, Levine PB (2012). Why is the teen birth rate in the United States so high and why does it matter?. J Econ Perspect.

[19] Gideon R (2013). Factors associated with adolescent pregnancy and fertility in Uganda: analysis of the 2011 demographic and health survey data. Am J Sociol Res.

[20] Neal S, Ruktanonchai C, Chandra-Mouli V, Matthews Z, Tatem AJ (2016). Mapping adolescent first births within three East African countries using data from demographic and health surveys: exploring geospatial methods to inform policy. Reprod Health.

[21] Ruzibiza Y (2021). ‘They are a shame to the community … ’ stigma, school attendance, solitude and resilience among pregnant teenagers and teenage mothers in Mahama refugee camp, Rwanda. Glob Public Health..

[22] Munezero D, Bigirimana J (2017). Jont program “Menyumenyeshe” for improving sexual and reproductive health of adolescents and youth in Burundi.

[23] French H (2019). How the “joint program” intervention should or might improve adolescent pregnancy in Burundi, how these potential effects could be encouraged, and where caution should be given.

[24] Institut de Statistiques et d’Études Économiques du Burundi (ISTEEBU), Ministère de la Santé Publique et de la Lutte, contre le Sida (MSPLS), ICF International. Enquête Démographique et de Santé 2010. Bujumbura, Burundi: ISTEEBU, MSPLS, et ICF International.; 2012. 419. Available from: https://dhsprogram.com/publications/publication-FR253-DHS-Final-Reports.cfm

[25] Segamba L, Ndikumasabo V, Makinson C, Ayad M (1988). Enquête Démographique et de Santé au Burundi 1987.

[26] Croft TN, Marshall AM, Allen CK, Arnold F, Assaf S, Balian S (2018). Guide to DHS statistics.

[27] Dean AG, Sullivan KM, Soe MM (2013). OpenEpi: open source epidemiologic statistics for public health, version 3.01. www.OpenEpi.com, updated 2013/04/06.

[28] Sommet N, Morselli D (2017). Keep calm and learn multilevel logistic modeling: a simplified three-step procedure using Stata, R, Mplus, and SPSS. Int Rev Soc Psychol.

[29] Merlo J, Chaix B, Ohlsson H, Beckman A, Johnell K, Hjerpe P (2006). A brief conceptual tutorial of multilevel analysis in social epidemiology: using measures of clustering in multilevel logistic regression to investigate contextual phenomena. J Epidemiol Community Health.

[30] Tesema GA, Worku MG (2021). Individual-and community-level determinants of neonatal mortality in the emerging regions of Ethiopia: a multilevel mixed-effect analysis. BMC Pregnancy Childbirth.

[31] Teshale AB, Tesema GA (2020). Determinants of births protected against neonatal tetanus in Ethiopia: a multilevel analysis using EDHS 2016 data. Plos One.

[32] Tessema ZT, Tamirat KS (2020). Determinants of high-risk fertility behavior among reproductive-age women in Ethiopia using the recent Ethiopian demographic health survey: a multilevel analysis. Trop Med Health.

[33] Heck RH, Thomas S, Tabata L (2013). Multilevel modeling of categorical outcomes using IBM SPSS.

[34] Sommers M. Adolescents and violence: lessons from Burundi. Belgium: Belgique: Universiteit Antwerpen, Institute of Development Policy (IOB); 2013.

[35] Berckmoes L, White B (2014). Youth, farming and Precarity in rural Burundi. Eur J Dev Res.

[36] Tokindang J, Bizabityo D, Coulibaly S, Nsabimana J-C (2015). Profil et déterminants de la pauvret : Rapport de l’enquête sur les Conditions de Vie et des Ménages (ECVMB-2013/2014).

[37] Schwarz J, Merten S (2022). ‘The body is difficult’: reproductive navigation through sociality and corporeality in rural Burundi. Cult Health Sex..

[38] Cieslik K, Giani M, Munoz Mora JC, Ngenzebuke RL, Verwimp P (2014). Inequality in education, school-dropout and adolescent lives in Burundi.

[39] Arieff A. Burundi’s Electoral Crisis: In Brief. Washington, DC: Congressional Research Service; 2015.

[40] Westeneng J, Reis R, Berckmoes LH, Berckmoes LH. The effectiveness of sexual and reproductive health education in Burundi: policy brief. Paris: UNESCO; 2020.

[41] Nzokirishaka A, Itua I (2018). Determinants of unmet need for family planning among married women of reproductive age in Burundi: a cross-sectional study. Contracept Reprod Med.

[42] Hindin MJ, Kalamar AM, Thompson T, Upadhyay UD (2016). Interventions to prevent unintended and repeat pregnancy among young people in low-and middle-income countries: a systematic review of the published and gray literature. J Adolesc Health.

[43] Nove A, Matthews Z, Neal S, Camacho AV (2014). Maternal mortality in adolescents compared with women of other ages: evidence from 144 countries. Lancet Global Health.

[44] Olausson PO, Cnattingius S, Haglund B (1999). Teenage pregnancies and risk of late fetal death and infant mortality. BJOG.

[45] Islam MM, Islam MK, Hasan MS, Hossain MB (2017). Adolescent motherhood in Bangladesh: trends and determinants. Plos One.

[46] WHO. Preventing early pregnancy and poor reproductive outcomes among adolescents in developing countries: What the evidence says? Geneva: World Health Organization; 2011. https://www.who.int/publications-detail-redirect/9789241502214. Accessed 31 Aug 2022.

[47] WHO. WHO recommendations on adolescent sexual and reproductive health and rights. Geneva: World Health Organization; 2018. https://www.who.int/publications-detail-redirect/9789241514606.

[48] Darroch JE, Woog V, Bankole A, Ashford LS, Points K (2016). Costs and benefits of meeting the contraceptive needs of adolescents.

[49] Isteebu. Recensement Général de la Population et de l’Habitat au Burundi en 2008. Bujumbura, Burundi: Institut de Statistiques et d’Études Économiques du Burundi; 2008. Available from: https://www.isteebu.bi/rgph-2008/

[50] Sully EA, Atuyambe L, Bukenya J, Whitehead HS, Blades N, Bankole A (2018). Estimating abortion incidence among adolescents and differences in postabortion care by age: a cross-sectional study of postabortion care patients in Uganda. Contraception.

[51] Sully E, Dibaba Y, Fetters T, Blades N, Bankole A (2018). Playing it safe: legal and clandestine abortions among adolescents in Ethiopia. J Adolesc Health.

[52] DHS Program (2020). The DHS Program - Request Access to Datasets. The Demographic and health surveys Program.

